# Managing aggressive vertebral hemangiomas with combination therapy: a case report and literature review

**DOI:** 10.3389/fonc.2025.1632237

**Published:** 2025-10-22

**Authors:** Zetao Jia, Jianhu Zheng, Xu Zhong, Xin Xiu, Aokang Xie, Guoyan Liu, Yungang Chen

**Affiliations:** ^1^ Rugao Hospital of Traditional Chinese Medicine, Nantong, China; ^2^ Wangjing Hospital, China Academy of Chinese Medical Sciences, Beijing, China; ^3^ Weifang Hospital of Traditional Chinese Medicine, Weifang, China; ^4^ Shandong University of Traditional Chinese Medicine, Jinan, China; ^5^ Affiliated Hospital of Shandong University of Traditional Chinese Medicine, Jinan, Shandong, China

**Keywords:** aggressive vertebral hemangioma, combination therapy, arterial embolization, vertebroplasty, total laminectomy with spinal canal decompression

## Abstract

Vertebral hemangiomas (VH), the most prevalent benign spinal tumors, often remain asymptomatic for many years or even a lifetime. Symptomatic cases are rare, constituting only 0.9%–1.2% of cases. Approximately 55% of symptomatic VH cases present with pain as the sole manifestation, whereas the remaining 45% exhibit aggressive characteristics and are classified as aggressive vertebral hemangiomas (AVH). AVH may invade the spinal canal and/or paravertebral space, potentially causing spinal cord compression and nerve damage, thus requiring active treatment. Despite various treatment options available for AVH, a consensus on the optimal therapeutic strategy is yet to be established owing to its rarity. We report a case of AVH who presented with symptoms of spinal cord compression treated using combination therapy of preoperative arterial embolization, intraoperative vertebroplasty, and total laminectomy with spinal canal decompression. This approach was tailored to the patient’s poor physical condition and yielded satisfactory clinical outcomes at 12-month follow-up. Combination therapy maximized synergistic benefits and leveraged the advantages of each procedure, thus achieving enhanced therapeutic effects and reducing risks. Given an aging population, tailoring combination therapy for AVH to individual patient characteristics merits broader clinical adoption.

## Introduction

Vertebral hemangiomas (VH) are the most common benign spinal tumors, with an incidence of approximately 10%–26% (1). Although VH can develop at any age, most lesions manifest after the age of 40 or 50 years, with a slight female predominance and a male-to-female incidence ratio of approximately 1:1.3–1:2.25. VH most frequently affects the thoracic spine and typically involve a single vertebra (2). Most cases remain asymptomatic throughout life (Enneking stage 1), with only 0.9%–1.2% becoming symptomatic. Among symptomatic cases, approximately 55% present solely with pain (Enneking stage 2), while the remaining 45% exhibit aggressive behavior and are classified as aggressive vertebral hemangiomas (AVH, Enneking stage 3) (1). AVH can invade the spinal canal and/or paravertebral space, potentially causing spinal cord compression and nerve damage, thus requiring active treatment. Although various treatment options are available for AVH, there is no established consensus on the optimal therapeutic strategy owing to its rarity (3). Herein, we present a case of a patient with AVH presenting with symptoms of spinal cord compression who was admitted to our hospital.

## Case presentation

A 66-year-old female was admitted to our hospital in April 2021 presenting with progressively worsening pain and numbness in the lower back, which began after exertion 1 year earlier, and in both lower limbs since 6 months prior without any obvious triggers. The patient reported pain and numbness in the lower back radiating to the posterior and lateral aspects of both thighs, with exacerbation upon activity, alleviation upon rest, and frequent urination.

**Figure 1:**
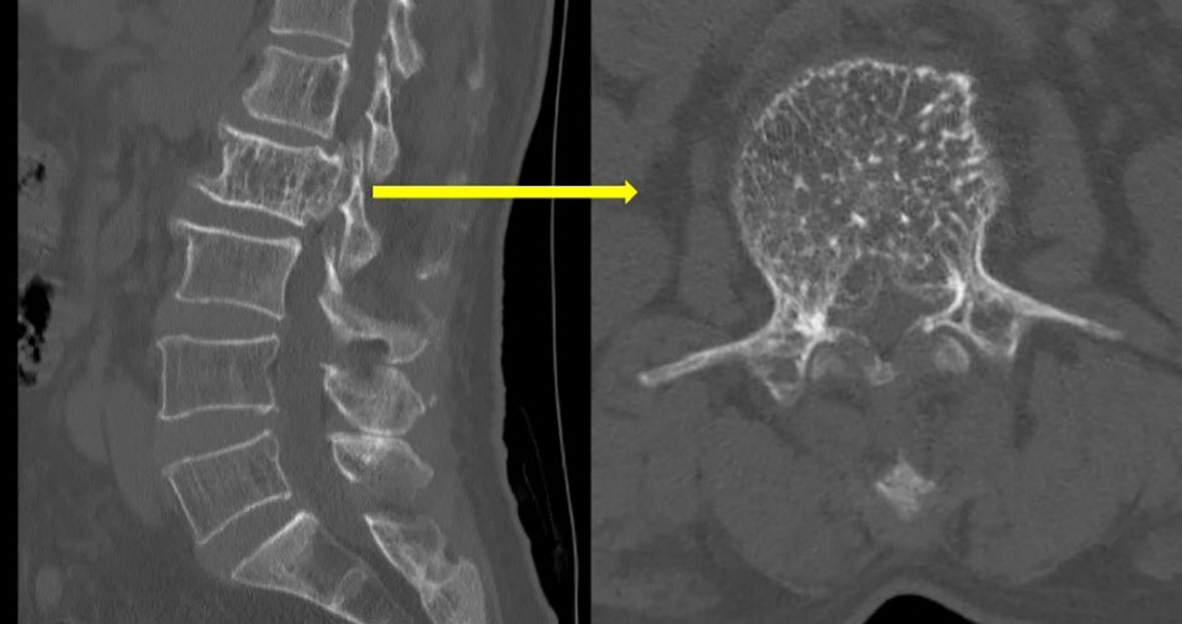
Preoperative lumbar CT revealed honeycomb and fence-like changes.

**Figure 2:**
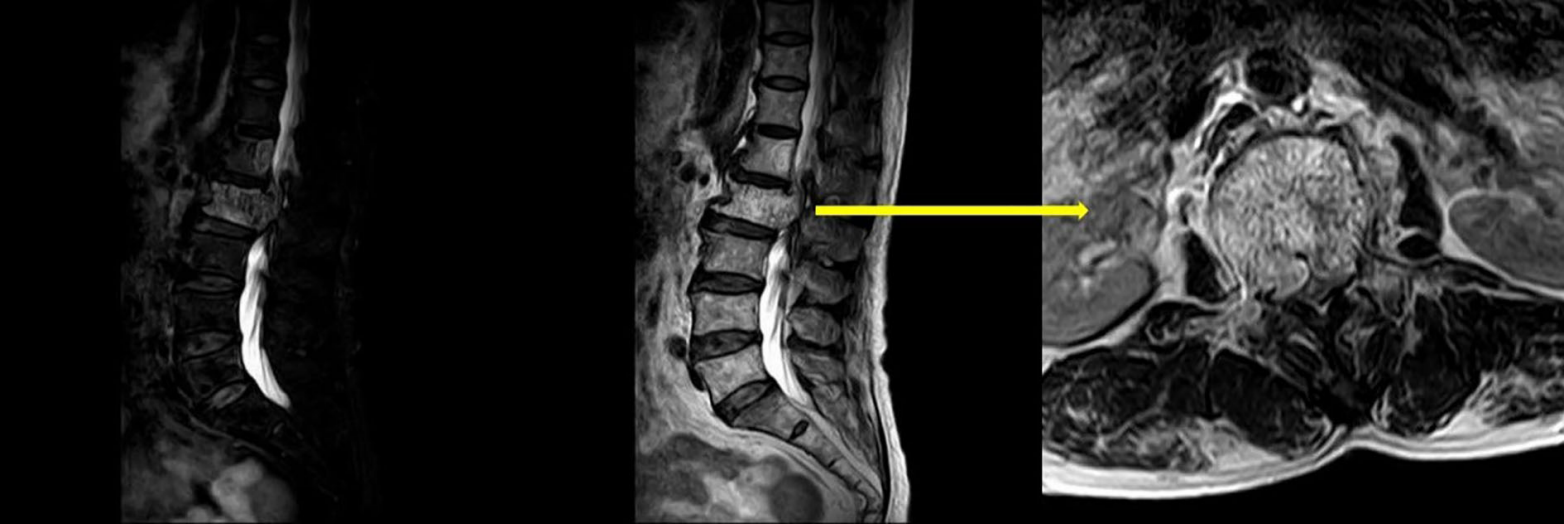
Preoperative lumbar MRI revealed that AVH had invaded the spinal canal and was compressing the spinal cord.

Upon physical examination, tenderness and percussion pain were observed in the L1 and L2 spinous processes, with radiating pain in both lower limbs. Bilateral iliopsoas muscle strength was graded as IV. The strength of the other muscle groups remained normal. The lumbar extension test was positive, and bilateral Babinski signs were negative. The mean preoperative visual analog scale (VAS) was 8. Preoperative lumbar CT revealed a mass with abnormal density in the L2 vertebral body and posterior elements, with vertebral body deformation and spinal stenosis, and within the L1 vertebral body and left posterior elements (**Figure 1**). Preoperative lumbar MRI revealed patchy T1 hypointensity and T2 hyperintensity on the left side of the L1 vertebral body and posterior elements, with high signal on fat-suppressed sequences. The L2 vertebral body was slightly flattened, with posterior bulging of the vertebral body margin. The vertebral body and bilateral posterior elements demonstrated T1 hypointensity and T2 hyperintensity, with high signal on fat-suppressed sequences, and the corresponding spinal canal was significantly compressed, narrowed, and deformed. Heterogeneous signals were observed within the spinal canal at the L1-L2 level, with compression and displacement of the cauda equina (**Figure 2**). Preoperative lower-limb electromyography was performed to further quantify the degree of bilateral lower extremity neurological dysfunction and to assist in differential diagnosis. The results revealed reduced motor conduction wave amplitude of the bilateral common peroneal nerves, a lack of definite waveforms induced by the right common peroneal nerve F-wave, and abnormal spontaneous electrical activity in the bilateral gastrocnemii, right tibialis anterior, and tibialis posterior muscles, with partial widening of the Motor Unit Potential duration in the right tibialis posterior muscle. The preoperative diagnosis was AVH at L1 and L2 (Enneking stage 3).

**Figure 3:**
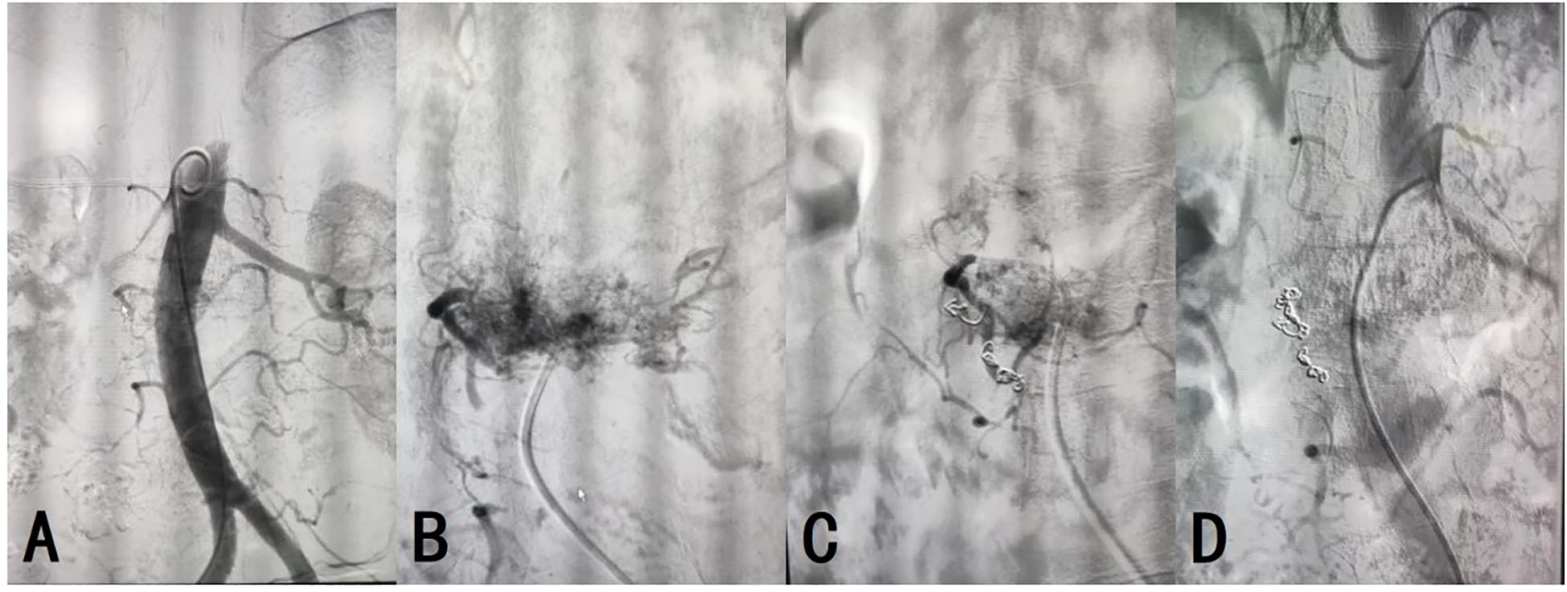
**(A)** Technical success of the arterial angiography was achieved. **(B–D)** The embolization of AVH was successful, with resolution of the abnormal vascular staining.

On day 2 post-admission, aortic and lumbar artery angiography and embolization were performed under local infiltration anesthesia. The patient was placed in the supine position and the surgical area was routinely disinfected. Using the Seldinger technique, the right common femoral artery was retrogradely punctured and a 6F catheter sheath was inserted. A 0.035 guidewire and a 5F pigtail catheter were used to guide the catheter into the proximal abdominal aorta. Angiography revealed abnormal abdominal aorta staining at L2, suggesting a hemangioma approximately 5×6 cm in size, primarily fed by the right first lumbar artery. Following C2 catheter, MPA catheter, and 0.035 guidewire entry, a microcatheter and microguidewire were introduced into the right first lumbar artery to introduce five 3×20 mm coils and two 5×20 mm coils for embolization. Follow-up angiography confirmed the disappearance of the abnormal right first lumbar artery staining and successful embolization (**Figure 3**). The guidewire and catheter sheath were withdrawn, and the puncture site was closed using a vascular closure device with local pressure dressing. The operation was uneventful, and the patient returned to the ward in good condition.

**Figure 4:**
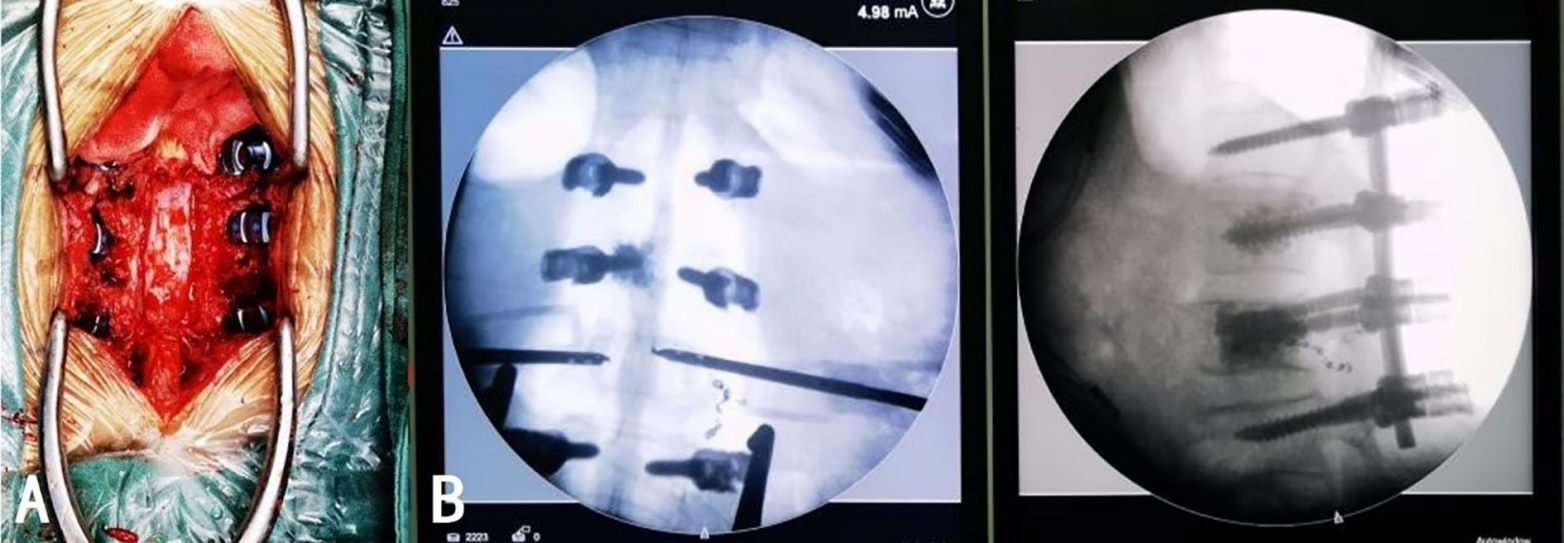
The position of the internal fixation under intraoperative direct vision **(A)** and fluoroscopy **(B)** was satisfactory. The distribution of the bone cement was appropriate without extravertebral leakage.

On day 6 post-admission, after the patient’s condition stabilized, L1 and L2 vertebroplasty, L1/2 and L2/3 total laminectomy, spinal canal decompression with bone grafting, and internal fixation were performed under general anesthesia. The patient was placed in the prone position and C-arm fluoroscopy was used to locate and mark the T12, L1, L2, and L3 vertebrae. A posterior median longitudinal incision of approximately 20 cm was made, the skin, subcutaneous tissue, deep fascia, and supraspinous ligament were sequentially incised, and hemostasis was performed. The bilateral paraspinal muscles were retracted to expose the bilateral facet joints from T12 to L3. Pedicle screws were implanted on both sides of T12 and L3, and on the right side of L1. Customized pedicle screws with channels were implanted into the pedicles on the left side of L1 and both sides of L2. Fluoroscopy revealed satisfactory screw placement. Tissue samples were obtained through the pedicle screw channels using a biopsy needle and approximately 3 and 6 mL of bone cement was slowly injected into the L1 and L2 vertebral bodies through the pedicle screw channels, respectively, under fluoroscopic guidance, ensuring no extravertebral leakage. The biopsy needle was slowly removed after the bone cement hardened. The L1 and L2 spinous processes were removed, an ultrasonic bone knife and lamina rongeur were used to remove the L1/2 and L2/3 laminae, and the removed laminae were trimmed into bone fragments for grafting. A pre-bent titanium rod was connected to the previously implanted pedicle screws, and the prepared bone fragments and artificial bone were impacted into the lateral posterior of T12, L1, L2, and L3. Fluoroscopy was utilized throughout the procedure to guide screw placement and verify the position of the rods and bone cement (**Figure 4**). After thoroughly rinsing with saline, hemostasis was achieved. The surgical instruments were counted to ensure that none were missing, the incision was sutured, and two drains were placed inside the wound. Intraoperative blood loss was approximately 500 mL, and 400 mL of type O Rh-positive plasma was transfused. The procedure was deemed successful. The patient was conscious, hemodynamically stable, and demonstrated good motor and sensory function in both lower limbs postoperatively.

**Figure 5:**
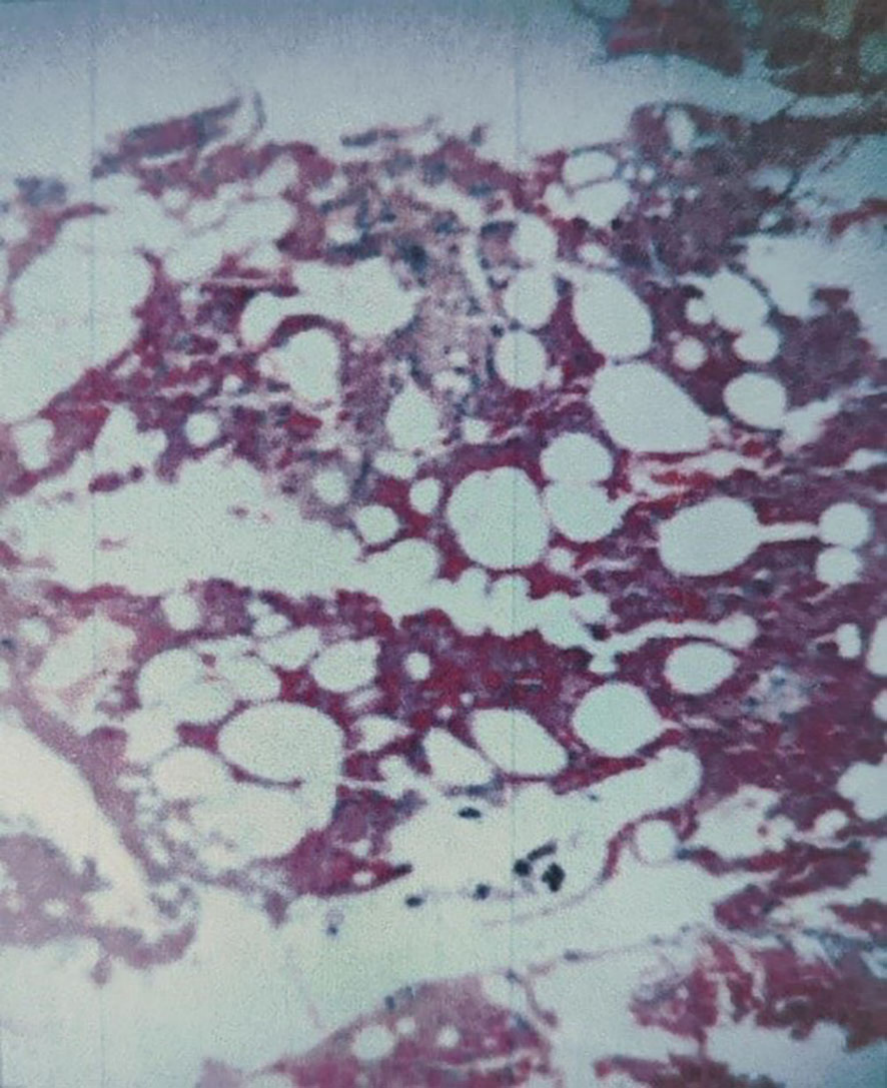
The pathological examination was consistent with AVH. Photomicrograph (H&E stain) revealed multiple thin-walled, dilated, blood-filled vascular channels scattered between bony trabeculae.

**Figure 6:**
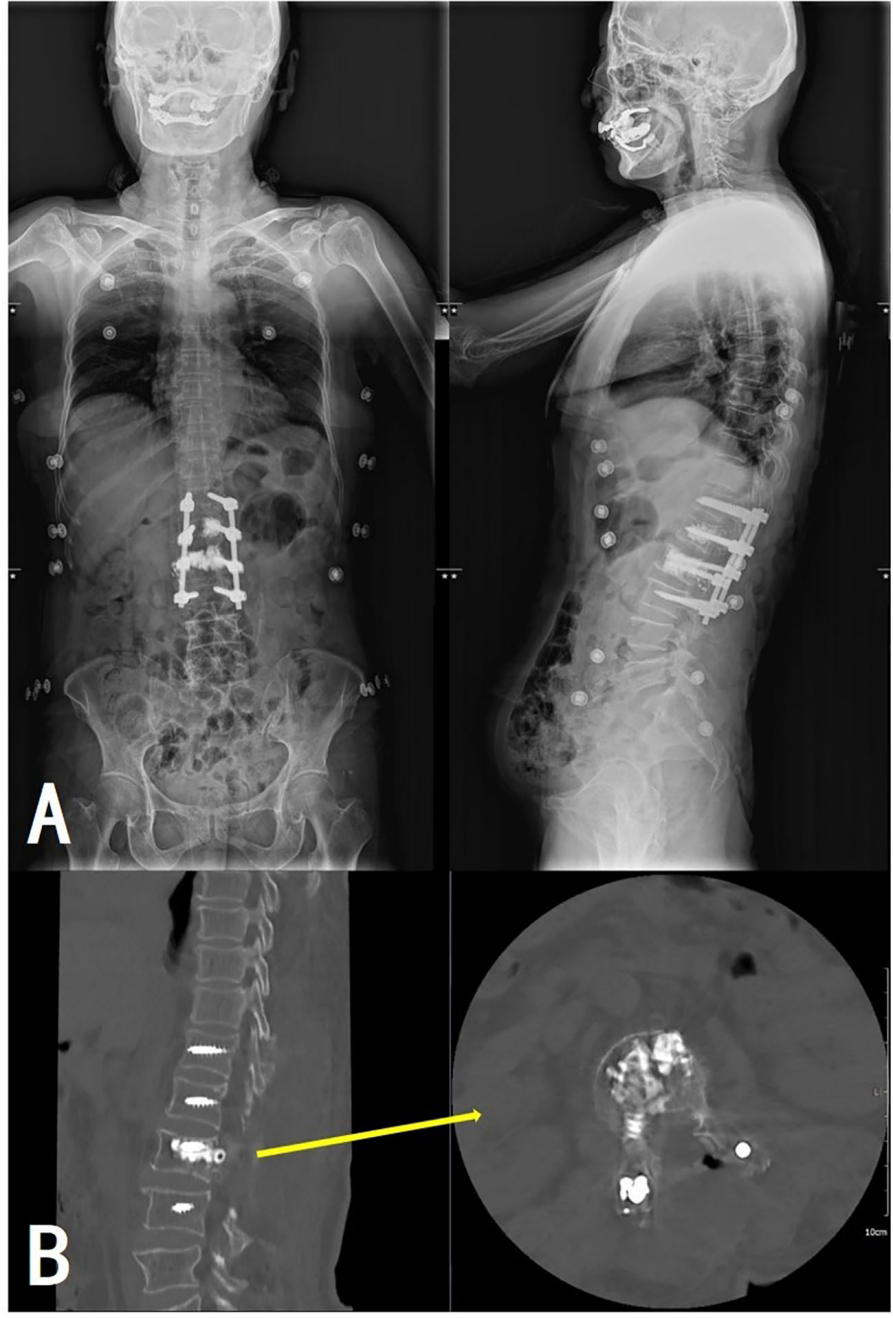
Postoperative full-length spinal X-ray **(A)** and lumbar CT **(B)** revealed satisfactory positions of the internal fixation and the bone cement without extravertebral leakage.

Pathological examination of tissue samples supported the diagnosis of AVH (**Figure 5**). The patient’s bilateral iliopsoas muscle strength recovered to grade V, and the pain and numbness symptoms were greatly alleviated, with a postoperative VAS of 2. Follow-up at 1.5, 6, and 12 months revealed good recovery of muscle strength and sensation, with no signs of recurrence (**Figure 6**).

## Discussion

Based on the clinical symptoms and imaging findings of this case, AVH initially presented with erosion of the L1 and L2 vertebral bodies and posterior elements, during which the patient experienced only low back pain (Enneking Stage 2). As AVH continued to erode the vertebrae, resulting in spinal canal narrowing and spinal cord compression, the patient developed symptoms of bilateral lower limb pain and numbness (Enneking Stage 3). The constellation of clinical symptoms — including posterior/lateral thigh radiculopathy (suggestive of L5/S1 involvement) and bilateral iliopsoas weakness (suggestive of L3/4 involvement) — is likely attributable to multi-level neural compression caused by the extensive AVH of L1 and L2. This understanding—that the patient’s complex neurological deficits originated from central compression at the L1-L2 level rather than from multiple isolated nerve root pathologies—was pivotal in formulating our treatment strategy. It directed our intervention towards the epicenter of the disease, AVH of L1 and L2, with the goal of alleviating the central canal compression, and addressing the root cause of the multi-level neurological symptoms.

VH was first reported in 1863 by Virchow et al., and its association with neurological symptoms was later described in 1895. In 1927, Makrykostas detailed how “ballooning” of the vertebrae or epidural extension of AVH could result in spinal canal stenosis and neurological symptoms (4). Despite this longstanding recognition, the optimal treatment strategy for AVH remains debated. Multiple therapeutic options, including radiotherapy, vertebroplasty, percutaneous ethanol ablation, arterial embolization, total/subtotal vertebrectomy, and total laminectomy with spinal canal decompression, have shown therapeutic efficacy, though each approach carries risks (**Table 1**).

**Table 1:** Summary of treatment modalities for AVH.

Treatment modality	Primary indications	Key advantages	Key limitations/Risks
Radiotherapy	Inoperable lesions, residual disease, adjuvant therapy	Non-invasive, effective for pain control	Risk of radiation myelopathy, necrosis, secondary malignancy; delayed effect
Vertebroplasty/Kyphoplasty	Painful lesions, vertebral body strengthening	Stabilizes vertebra, rapid pain relief, minimally invasive	Cement leakage (pulmonary embolism, nerve root compression)
Percutaneous Ethanol Ablation	Select vascular lesions	Direct ablation of lesion	High risk of complications (necrosis, collapse, neurological injury, cardiotoxicity)
Arterial Embolization	Preoperative adjunct, primary therapy for bleeding	Significantly reduces intraoperative blood loss	Non-target embolization, requires expertise, typically not standalone
Total Laminectomy/Decompression	Neurological deficit from posterior compression	Direct neural decompression, preserves anterior column	Does not address anterior vertebral body disease alone
Total/Subtotal Vertebrectomy	Extensive anterior/posterior involvement, failure of other treatments	Most complete lesion removal	High morbidity, technically demanding, requires reconstruction

### Radiotherapy

Radiotherapy, recognized as an effective treatment for AVH since the 1930s (3), reduces AVH volume by eliminating abnormal veins and capillaries and damaging the vascular endothelium over time (5). Studies from 1939 to 1985 reported improvement rates >60% in patients with AVH-induced neurological dysfunction (6, 7). However, the use of radiotherapy alone remains controversial. While it provides pain relief, it can cause radiation-induced tissue necrosis, pseudoarthrosis, skin complications, and malignancy (8, 9). It is often reserved for inoperable cases, residual lesions, or as an adjuvant therapy (3).

### Vertebroplasty

Since the late 1980s, vertebroplasty has been used to treat VH (10). The exothermic polymerization of bone cement not only obliterates hemangioma cells, but also induces necrosis of nerve endings, thereby alleviating pain symptoms. The curing process of bone cement augments vertebral body strength, enhancing its stability and reducing pain symptoms (11). Guarnieri et al. (12) reported 24 cases of VH treated with vertebroplasty (6 of which were aggressive) and reported no recurrence during a 4-year follow-up. During open surgery for AVH, vertebroplasty has also been shown to reduce intraoperative bleeding and complications (13). Furthermore, the risk of pulmonary embolism resulting from bone cement leakage into the paravertebral venous plexus during vertebroplasty must be carefully considered, particularly in AVH, which is a vascular anomaly. In this case, preoperative embolization significantly reduced vascular flow within the lesion, thereby mitigating the risk of bone cement leakage. Additionally, slow injection of high-viscosity cement under continuous fluoroscopic guidance further enhanced procedural safety.

### Percutaneous ethanol ablation

Percutaneous ethanol ablation to treat AVH was first proposed in 1994 (14). Despite showing early promise, with complete lesion resolution on postoperative angiography in 11 patients, long-term follow-up data are lacking (15), and many complications have been reported, such as osteonecrosis, vertebral collapse, transient neurological deterioration, spinal cord injury, hemodynamic instability, and cardiac arrest (16–18). Thus, percutaneous ethanol ablation is gradually fading out of mainstream practice.

### Arterial embolization

Given the highly vascular nature of AVH, one of the primary surgical risks is uncontrolled massive bleeding. In the 1970s, angiography was used to both confirm the presence of AVH and guide feeding artery embolization to reduce intraoperative bleeding (19). Subsequent studies have found that preoperative embolization effectively reduces intraoperative blood loss and minimizes perioperative complications (20) Cotten et al. (21) reported that combining preoperative arterial embolization with intraoperative vertebroplasty can further reduce intraoperative bleeding.

### Surgical management

Common surgical approaches for AVH include total/subtotal vertebrectomy and total laminectomy with spinal canal decompression. In a retrospective study of 10 patients with AVH who underwent total vertebrectomy and preoperative feeding artery embolization, Acosta et al. (22) reported no recurrence over a mean follow-up period of 2.42 years. Similarly, Djindjian et al. (23) reported complete symptomatic relief and no recurrence during the 6-year follow-up in a patient who received auxiliary radiotherapy after arterial embolization and total laminectomy with spinal canal decompression. However, total vertebrectomy is technically challenging, with complication rates as high as 36.3% (24). Hence, clinicians must comprehensively evaluate the patient’s condition to select an appropriate surgical method.

In our case, preoperative angiography confirmed the diagnosis of AVH and facilitated feeding artery embolization to prevent massive intraoperative bleeding. Intraoperative vertebroplasty was performed to inactivate the AVH using thermal necrosis, enhance vertebral strength, and ultimately alleviate the patient’s pain symptoms, while minimizing bleeding during the procedure. Considering the patient’s age and poor physical condition, total laminectomy with spinal canal decompression was selected. By removing the spinous processes and bilateral laminae of L1 and L2, the compressed spinal cord was indirectly decompressed by expanding spinal cord volume behind the lesion. Unlike total/subtotal vertebrectomy, this procedure preserved the anatomical structure of the anterior and middle vertebral columns, maintained spinal column stability, reduced surgical time, and lowered the risk of neurological injury. Moreover, this procedure reduced intraoperative bleeding, which lessened the physical burden on the patient, reduced the incidence of complications, and facilitated postoperative recovery. The patient showed no recurrence at 1.5, 6, and 12 months follow-up. If recurrence or residual lesions are suspected upon further follow-up, we recommend radiotherapy or total vertebrectomy with spinal reconstruction using a titanium mesh cage for complete removal. Although the individual components of this combination therapy are not novel, and some combinations have been reported in previous case reports (25–27), they were typically employed as concurrent procedures rather than a predefined treatment strategy. To our knowledge, this is the first case report to formally articulate and evaluate ‘combination therapy’ as a cohesive and rationalized treatment paradigm for AVH.

## Conclusion

This case incorporated combination therapy tailored to the individual patient characteristics, comprising selected preoperative arterial embolization, intraoperative vertebroplasty, and total laminectomy with spinal canal decompression for AVH treatment. Preoperative embolization and intraoperative vertebroplasty significantly reduced intraoperative blood loss. Vertebroplasty not only enhanced vertebral body strength but also contributed to lesion eradication. The limited decompression and fusion surgery minimized surgical trauma while effectively preventing adverse outcomes such as vertebral collapse and kyphotic deformity. Combination therapy maximized synergistic benefits and leveraged the advantages of each procedure, thereby achieving improved therapeutic outcomes while reducing risks. Given an aging population, tailoring combination therapy for AVH to individual patient characteristics merits broader clinical adoption.

## Data Availability

The original contributions presented in the study are included in the article/supplementary material. Further inquiries can be directed to the corresponding author.
